# The P.E.A.N.U.T. Method: Update on an Integrative System Approach for the Treatment of Chronic Otitis Media with Effusion and Adenoid Hypertrophy in Children

**DOI:** 10.3390/antibiotics10020134

**Published:** 2021-01-30

**Authors:** Henrik Szőke, Márta Maródi, Jan Vagedes, Balázs Székely, István Magyarosi, Adél Bedő, Veronika Fellegi, Krisztina Somogyvári, Péter Móricz

**Affiliations:** 1 Department of IM, Faculty of Health Sciences, University of Pécs, 7623 Pécs, Hungary; 2 Doctoral School, Faculty of Health Sciences, University of Pécs, 7623 Pécs, Hungary; 3 Department of Otolaryngology, Szt. Borbála Hospital, 2800 Tatabánya, Hungary; doktormarodi@gmail.com; 4 Department of Neonatology, University Children’s Hospital Tubingen, 72016 Tubingen, Germany; j.vagedes@arcim-institute.de; 5 ARCIM Institute (Academic Research in Complementary and Integrative Medicine), 70794 Filderstadt, Germany; 6 Cello Health Insight, Advanced Analytics Manager, London EC1M 7AP, UK; szekelyb.ppke@gmail.com; 7 Department of Otolaryngology, Balassa János Hospital, 7100 Szekszárd, Hungary; mistvan19@yahoo.com (I.M.); moricz.peter@tmkorhaz.hu (P.M.); 8 Doctoral School, Faculty of Medicine, University of Pécs, 7622 Pécs, Hungary; 9 Outpatient Clinic, 8000 Székesfehérvár, Hungary; dr.adel.bedo@gmail.com (A.B.); fvera@datatrans.hu (V.F.); 10 Department of Otorhinolaryngology and Head-Neck Surgery, Clinical Center, University of Pécs, 7621 Pécs, Hungary; dr.somogyvari.k@gmail.com

**Keywords:** adenoid hypertrophy, analgesics, antibiotics, children, chronic otitis media with effusion, integrative therapy, surgical interventions

## Abstract

Background and objectives: Based on our previous single-center study on optimization of treatment of chronic otitis media with effusion (COME) and adenoid hypertrophy (AH) in children using a noninvasive system approach to lower the necessity of antibiotics, analgesic use, and surgical interventions, we proceeded to perform a multicenter investigation in an outpatient setting. The purpose of the previous prospective study in 2013–2015 was to compare outcomes in the treatment of COME and AH using the noninvasive multimodal integrative method (IM) versus conventional treatment practice (COM). Materials and Methods: In this paper, we retrospectively analyze the data of patients treated with the integrative method between 2017 and 2020 in a multicenter setting and compared the outcomes with data from 2013–2015 in order to evaluate generalizability. In both periods, all eligible and willing participants were included and treated with the IM protocol under real-life conditions. The treatment involved pneumatization exercises, education, an antiallergic diet, nasal hygiene, useful constitutional therapy, and thermal interventions (P.E.A.N.U.T.). A total of 48 versus 28 patients, aged 1–8, were assessed, presenting with COME and AH, with moderate to severe hearing impairment at entry. Results: The significant improvement found in both audiometric measures (intact hearing) and tympanometric measures (normal A-type curve) was similar in both datasets with respect to conventional treatment. The new data confirms that the P.E.A.N.U.T. method results in a significant reduction of antibiotics, analgesic use, and surgical interventions. Conclusion: In this multicenter trial, we confirm the effectiveness of the noninvasive system approach for the treatment of COME in lowering the need for antibiotics and analgesic use and elective surgery. This could be especially important with respect to a generally observed increase in antibiotic resistance. The method is easy to perform in different clinical settings and is effective, safe, and well-tolerated.

## 1. Introduction

COME and AH are common entities in the pediatric population. The risk factors are diverse, including wet and cold climates, age (1–6 years), number of siblings, passive smoke, swimming pool visits, and allergies [1].

The microbiome and biofilm play important roles in modulating immune homeostasis and disease susceptibility; the nasopharyngeal microbiome is also involved in the pathogenesis of otitis media [2,3,4,5].

COME is diagnosed when typical clinical symptoms are present with audiometric and/or tympanometric measurements. An ear/nasopharynx endoscopy may be performed.

The longer COME persists, the higher the rate of complications and the lower the rate for natural resolution.

Conventional treatment of AH and COME, mainly surgical, remains an option after a period of watchful waiting—with local warmth and decongestants, mucolytics [6,7,8,9].

According to the latest guidelines, the routine use of antibiotics, local steroids, decongestants, and antihistamines is not effective and recommended, although often prescribed for the treatment of COME [10]. 

The period of watchful waiting opens a therapeutic window for noninvasive methods, e.g., the P.E.A.N.U.T. method.

We have previously shown, in a pilot single-center study, that this approach could be effective.

In this multicenter study, covering a larger population over an extended time period, we hypothesize that using the P.E.A.N.U.T. method in the treatment of early stages of COME, associated with episodes of recurrent acute otitis media (rAOM), lowers the need for antibiotics and analgesic use and surgical interventions. Additionally, we hypothesize that this multimodal approach represents a generalizable, safe, and effective alternative to the usual approaches.

## 2. Materials and Methods

### 2.1. Study Design

The original prospective pilot study, between 2013 and 2015, was a single-center investigation with nonrandomized group assignments and a descriptive statistical comparison of outcomes. The details of study design, treatment, and results were published in 2016 [11].

In the present study, during the time period of 2017–2020, more patients (*n* = 48) were treated with the IM protocol (P.E.A.N.U.T.) in five different outpatient clinics. All eligible patients were included in the study. The study design remained the same, as previously described [11]. In this paper, we describe a retrospective analysis of both 2013–2015 and 2017–2020 datasets.

For spontaneous regression rates for COME, historical data were used as control.

### 2.2. Patients

All 1- to 8-year-old patients who met inclusion criteria were eligible to participate in the study if symptoms persisted for longer than 3 months. COME and AH were diagnosed by an otolaryngologist. Inclusion criteria included moderate (30–40 dB on the less affected ear) or severe (over 40 dB on the less affected ear) hearing loss and at least one abnormal tympanometric finding during the first three visits.

Children with anatomical abnormalities, concurrent illnesses, congenital syndromes, and previous otolaryngologic surgical interventions were excluded from the study.

It was not considered ethical to leave patients without any treatment, because patients with COME and moderate-to-severe hearing impairment for a “watchful waiting period” of more than 3 months were included in the studies.

The follow-up period lasted for 200–400 days, consisting (preferably) of monthly visits.

### 2.3. Treatment

The P.E.A.N.U.T. method involves pneumatization exercises, education, an antiallergic diet, nasal hygiene, useful constitutional therapy, and thermal interventions, as published in detail earlier [11]. All patients included in the P.E.A.N.U.T. group were treated with the entire P.E.A.N.U.T. protocol for the entire duration of the study.

#### 2.3.1. P for Pneumatization Exercises

I. Passive techniques without pressure, such as Eustachian tube rehabilitation therapy (ETRT) [12];

II. Low-pressure exercises (ca. 0–50 mmHg);

III. Middle-pressure exercises (ca. 50–100 mmHg);

IV. High-pressure autoinsufflation exercises (ca. 100–150 mmHg);

(a) autoinflation balloon techniques [13,14,15];

(b) the Valsalva maneuver;

(c) the Misurya maneuver.

#### 2.3.2. E for Education of Parents and Patients

As underlined by prior data, health literacy and practical instructions regarding treatment implementation enhance compliance and efficacy [16]; therefore, a detailed education program was performed using the motto: “Be exact, simple, and easy to understand”.

#### 2.3.3. A for Antiallergic Diet

In the case of elevated specific IgE levels and/or IgG positivity to beta-lactoglobulin, albumin, or casein, patients were asked to adhere to complete elimination of milk and dairy products during the study period [17]. All other patients without definite allergies were asked to comply with a partial restriction of milk and dairy products, limited to 150 g/day. In the study population, 15% of the patients adhered to a complete elimination diet and 85% to a partial elimination diet of cow milk and dairy products.

#### 2.3.4. N for Nasal Local Preparations

A nasal spray with Cydonia fructibus glycerinum extractum (APC 3.0; 9% volume) and citrus lemon fruct. (2% volume) in a 1% volume NaCl solvent, 4–5 times per day to both nostrils, was used on the patients.

The effect of this combination is based on decongestant, astringent [18] and immunomodulatory [19,20] properties, i.e., anti-inflammatory [21], antibacterial [22], and antiallergic properties [23,24,25].

A key feature in the application of the nasal spray was a horizontal application so that the adenoid and the entrances of the auditory tubes could be reached effectively. Nasal preparations were applied with standard nasal spray nozzles without any additional pressure (as it is necessary to avoid the risk of injecting mucus into the middle ear through the Eustachian tube during the application of sprays) or autoinflation methods.

#### 2.3.5. U for Useful Constitutional Medication

In our study, in this regard, Berberis/Quarz Glob. (WALA) was used as the single intervention [26]. 

#### 2.3.6. T for Thermal Interventions (Externally Applied Warm)

- Local intervention with infrared light or warming cap, 2- to 3-times per week for few minutes, on the ears.

- Systemic measures such as avoiding cold areas on the patient’s body and daily warm (ca. 40–45 °C) footbaths with 1–1.5% NaCl and Zingiber officinale pulvis [27].

### 2.4. Outcome Variables

Primary outcome variables are detailed in Table 1.

Secondary outcome variables are detailed in Table 2.

### 2.5. Statistical Analysis

Statistical evaluation for the datasets was performed using IBM SPSS Statistics 22 software and Microsoft Excel. The following tests were applied, where appropriate: test for median differences, Chi-squared test, Mann–Whitney test, and Fischer’s 2-sided exact test. For all analyses, *p* ≤ 0.05 was considered significant.

### 2.6. Human Research Ethics Committee

Ethics Committee approval was granted (IV-2425–5/EKU), all patient caregivers gave written informed consent, and the study was performed according to the Declaration of Helsinki.

## 3. Results

### 3.1. Baseline Characteristics

The baseline characteristics for both years are detailed in Table 3.

In 2015 and 2018, 28 and 48 patients, respectively, aged 1–8 years at entrance, were evaluated. Hearing loss was measured audiometrically at baseline. It was (minimal + moderate + severe) higher in 2015 than in 2020, with different distributions. The other baseline parameters were not significantly different. Subjective and objective parameters were congruent.

No patient had to be excluded; no patient was lost to follow-up during the observation period of 2017–2020.

### 3.2. Primary Outcomes Results

Primary outcomes results are listed in Table 4.

### 3.3. Frequency of Antibiotic Use

In the 2013–2015 study group, antibiotic use in the foregoing year before entry into the study was regarded as the baseline, which was similar in both groups. It was 1.57 treatments/year/patient in the IM group and 1.33 treatments/year/patient in the COM group (Mann–Whitney test, *p*-value 0.833). In the 2017–2020 study group (P.E.A.N.U.T., e.g., IM), the baseline value was similar (1.7 treatments/year/patient in the foregoing year before entry (Mann–Whitney test, *p*-value ≤0.71). These values are in concordance with previous literature for conventional setting treatments [28,29].

During the observed study periods, a significant difference was detected.

In the conventional treatment group of 2013–2015, antibiotic use remained high (1.4 treatments/year/patient), similar to the baseline value (1.33 treatments/year/patient).

In the 2013–2015 study, for the IM group, this value was significantly lower (0.21 treatments/year/patient; Mann–Whitney test, *p*-value 0.000). In 2020, with the P.E.A.N.U.T. protocol (IM), a similarly low antibiotic use was detected (0.48 treatments/year/patient; Mann–Whitney test, *p*-value 0.001). The Mann–Whitney test for the IM groups of the two periods showed a consistent size of the effect (*p*-value 0.545).

### 3.4. Frequency of Analgesic or Antipyretic Medication 

The frequency of analgesic or antipyretic medication (local and systemic) was significantly lower (*p*-value of *t*-test: 0.006 in 2015, 0.001 in 2020) than at baseline and also much lower during both periods compared to the conventional treatment group.

### 3.5. Number of Surgical Interventions 

The number of adenoidectomy + myringotomy + ventilation tube insertion was not significantly different in 2015 compared to 2020. They were less common during both periods compared to the usual treatment settings.

### 3.6. Improvement in Tympanometric and Audiometric Measurements 

**Figure 1:**
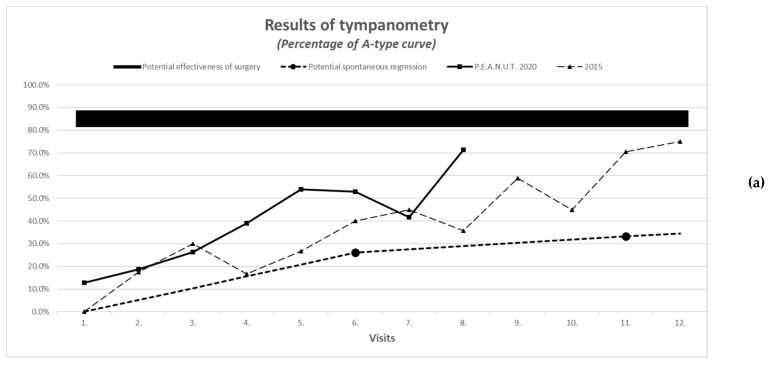

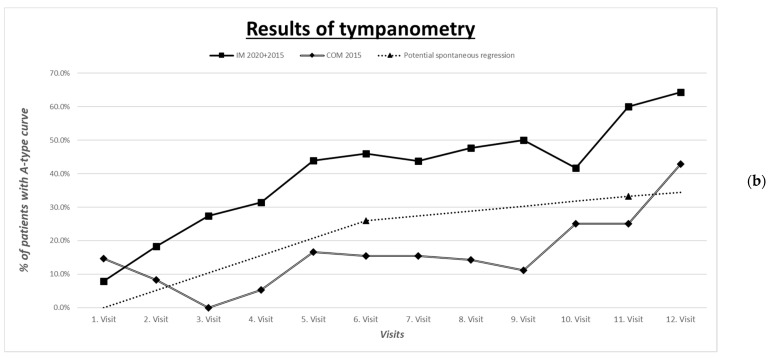
(**a**) Improvement in tympanometric measurement IM (2015 vs. 2020). (**b**) Improvement in tympanometric measurement (COM 2015 vs. IM 2015 + 2020).

**Figure 2:**
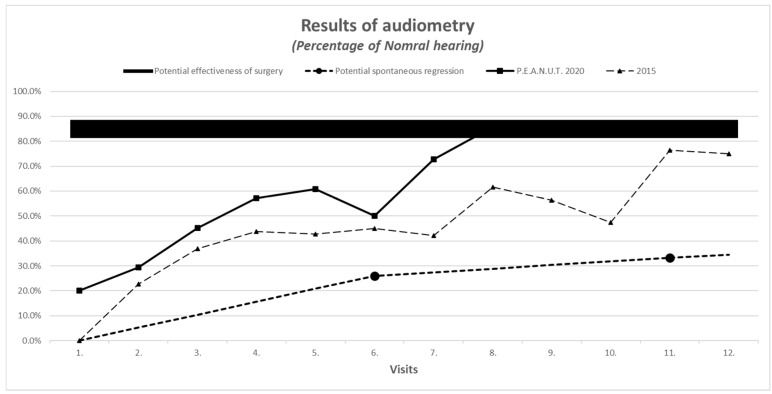
Improvement in audiometric measurement.

The improvement in A-type curve with normal pressure (see Figure 1a,b) and intact hearing (see Figure 2) were more than expected with natural resolution. Cramer’s V-test for improvement in A-type tympanometric findings showed a medium effect size in the majority of the cases during the follow-up.

### 3.7. Results of Secondary Outcomes

The results are listed in Table 5.

The number of patients with acute otitis media and the frequency of acute otitis media/year/patient during the observation period was reduced from 1.5–1.6 at baseline to 0.71–0.86 during the period of therapy.

Adherence to the treatments was good and not significantly different in the different periods and between the intervention groups. The median duration of follow-ups was not significantly different in 2020 compared to 2015.

The length of the therapeutic period was significantly shorter in 2020, so the statistically evaluated number of follow-ups was also lower in 2020 (*n* = 8) than in 2015 (*n* = 12).

Adverse reactions were similarly low compared to historical data.

**Figure 3:**
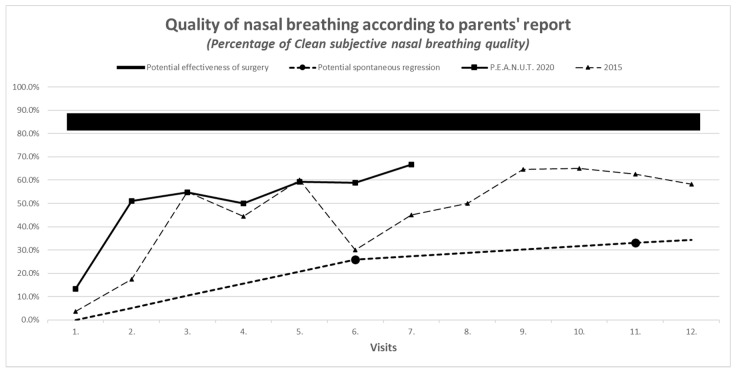
Parents’ subjective reports on nasal breathing.

**Figure 4:**
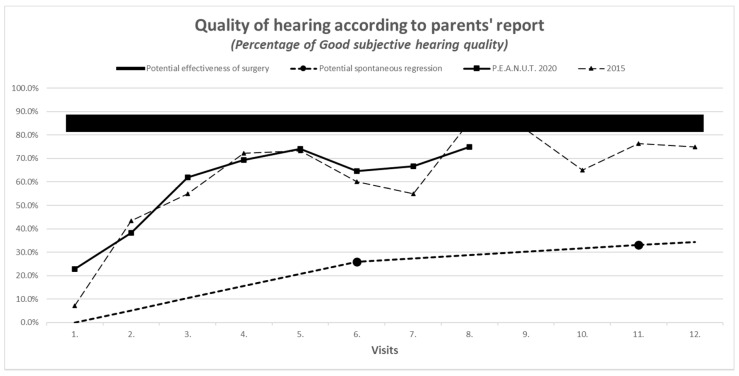
Parents’ subjective reports on hearing.

Parent reports on treatment outcome, as described by the scale for nasal congestion (1 = none, 2 = moderate, 3 = severe), was concordant with objective measures on audiometry and tympanometry and had similar improvement in 2020 compared to 2015 (see Figure 1, Figure 2, Figure 3 and Figure 4).

Parent reports on treatment outcomes, using the hearing scale (1 = good, 2 = moderate, 3 = bad) was concordant with objective measures on audiometry and tympanometry and had similar improvement in 2020 compared to 2015 (see Figure 1, Figure 2, Figure 3 and Figure 4).

## 4. Discussion

### 4.1. Main Findings

The two compared studies showed similar baseline characteristics of patient measures regarding mean age, number of previous treatments, and severity of the illness as determined by subjective and objective measures, except for distribution of audiometric findings.

Primary outcomes of the study in the 2017–2020 treatment period confirmed the results from the previous pilot study, which had given a preliminary indication that the integrative approach might be effective in improving the symptoms of COME and might have the potential to minimize the antibiotic prescription rate and the need for surgery. These results remained significant and stable over 7 years of observation, with more practitioners and patients involved.

Secondary outcome measures suggest that the P.E.A.N.U.T. method requires more effort in terms of the need to implement a multimodal treatment, with stricter adherence and more patient education. On the other hand, costs and adverse reactions can be reduced. The multimodal method has the additional benefit of creating more opportunities for a more personalized treatment plan and more active participation of caregivers and patients.

### 4.2. Additional Finding

The rate of cesarean section at birth was significantly lower in the integrative groups of 2015 and 2020 compared to the standard-care group of 2015 [11]. This may mirror the attitude of parents to surgical interventions, which was near the optimum of 10%, as per WHO recommendations.

This might also be influenced by the preoperative information given by the treating specialist and might imply the importance of health literacy and education in the informed decision process about different treatment strategies.

### 4.3. Strengths and Limitations

Numerous objective and subjective variables were evaluated. Due to a long follow-up period and relatively good adherence to follow-up visits, we were able to observe the clinical course closely and were well placed to distinguish between fluctuations in symptomatology and spontaneous remission (e.g., natural resolution) vs. resolution by treatment.

In terms of *selection bias,* we evaluated a good number of baseline parameters; these were not significantly different in 2015 vs. 2020, except for parents’ initial reports on nasal obstruction. The latter was unlikely to influence the assessment of efficacy. In terms of spontaneous resolution, with different treatment modalities, the prognostic factors remained similar. No attrition bias was detected in 2020 as no exclusion or loss of patient/dropout during the observation period occurred.

An *observation/reporting bias* was possible as, regarding audiometry, the worse ear result was involved in the statistical evaluation because subjective judgment by the caregivers implied the better ear. However, in many children, especially those under 4 years of age, it is not possible to obtain precise values for hearing loss in tonal audiometry. Just as different forms of audiometry are regarded as subjective measures in the literature, tympanometric measurement is generally accepted as the gold standard for objective investigation. To address this possible bias in the study, we evaluated the correlation of the improvement in tympanometric measures vs. audiometric values and tympanometric measures vs. subjective hearing reports by parents. All of these three measures were found to be correlated in all age groups in both studies, making it unlikely for this to be a manifestation of any observation bias. Additionally, as the observation period was between min. 200 and max. 400 days, almost all children passed the age limit of 4 years during the study period. So the proportion of cases where audiometric values were not feasible decreased toward the end of the observation period to a minimal level.

A *detection bias* was present as not enough data could be evaluated after the 8th visit in 2020, resulting in significantly less number of visits and a shorter length of observation period compared to 2015 due to the faster improvement of symptoms based on a more efficient implementation of the method by the physicians. In order to identify the impact of detection bias on the durability of the results, all patient data were assessed 6 months after the end of data collection with regards to primary and secondary outcome variables. Adenoidectomy had to be performed on only one patient. No significant relapse occurred during follow-up.

### 4.4. Interpretation, Relation to Previously Published Work

Integrative system approaches might result in a benefit in treating upper respiratory tract infections in children [30,31] and can reduce the need for antibiotic and analgesic use [32,33,34,35,36]. This study confirms previous data and extends them in terms of reducing the need for antibiotics and invasive interventions in the treatment of COME and AH [11].

Previous research has delivered evidence that nonsurgical treatment options for chronic OME and nasal balloon autoinflation (pneumatization exercises) have the most promising benefit on effect size. Although current scientific evidence suggests there may be a higher rate of short-term tympanometric cure than in controls, all published studies lacked intermediate and long-term follow-up and were investigated in largely selected secondary-care populations. Our study was conducted under the real-life conditions of primary care for over a whole year of observation to minimize this bias.

The data presented in Table 6 show the additional effect of the multimodal treatment compared to single-component interventions, including natural resolution.

All single interventions seemed to provide an additional effect, making it worthwhile to apply them multimodally [18,19,20,21,22,23,24,25,27]. This may make it difficult to sort out individual contributions to the outcomes. Further analysis of datasets with more patients is needed to detect the sufficient effect size for single interventions and patient subgroups. This study needs to be followed up by investigations involving a prospective randomized study design.

## 5. Conclusions

Regarding the hypothesis of the study, the use of the P.E.A.N.U.T. method in the treatment of COME, AH, and rAOM was able to reduce the need for antibiotic and analgesic use and surgical interventions in multicentered outpatient settings over a long period of time. It presents a low-cost, safe, and effective treatment that is easy to apply in any healthcare system. This could be especially important to the generally observed increase in antibiotic resistance.

Regarding indications for the outlined P.E.A.N.U.T. method, we suggest it in the following scenarios: in the case of fluctuating symptoms in the early stage of COME (Eustachian tube dysfunction); during the 3 months of watchful waiting (“active observation”) in COME, at the stage of glue ear; in the case of children with high risk for complicated runoff; if parents do not prefer or special circumstances exclude surgical interventions, so that a nonsurgical solution is sought.

There are clinical cases where conventional interventions may represent the best approach for treatment, including the need for surgical intervention. This is the case in patients with contraindications for the P.E.A.N.U.T. method. Contraindications include (1) advanced stage of COME (adhesive otitis media); (2) the presence of significantly hypertrophic adenoid vegetation, with signs of COME for more than 3 months; (3) any risk for complications such as severe hearing loss or sleep apnea; (4) developmental problems such as adhesions or cholesteatoma; (5) difficulties in communication with parents are present.

The results should be regarded as preliminary, pending larger studies that are able to differentiate between the benefit gained from single elements of the multimodal approach, with larger patient numbers and a prospective design.

## Figures and Tables

**Table 1:** Primary outcome variables.

(1) Number of invasive surgical interventions	Count
(2) Number of acute infection episodes needing antibiotic therapy	Number during the observation period
(3) Number of acute infection episodes needing analgesic therapy (local and systemic)	Number during the observation period
(4) Tympanometric measurement with evaluation of the worse ear	A-type curve: normal pressure C- or D-type curve: under- or overpressure B-type curve: low admittance
(5) Audiometric measurement with evaluation of the worse ear	Normal hearing: −10 to −20 dB Light hearing loss: −30 to −40 dB Middle hearing loss: −50 to −60 dB Severe hearing loss: >−60 dB

**Table 2:** Secondary outcome variables.

(1) Frequency of acute otitis media during the observed time period	Count
(2) Parents’ report on treatment outcome by subjective scales: (a) nasal congestion;(b) hearing	(a) 1 = no, 2 = moderate, 3 = severe (b) 1 = good, 2 = moderate, 3 = bad
(3) Adherence to therapy by subjective scale	1 = good, 2 = moderate, 3 = bad
(4) Median time of follow-ups	Days
(5) Mean number of days counted from the first visit during the follow-up period	Days
(6) Participation in the visits	Number of patients, %

**Table 3:** Baseline characteristics.

	2015	2020	*p*-Value
Number of patients evaluated	28	48	-
Mean baseline age in months	56.2	58.5	Mann–Whitney test 0.76
Tympanometry: abnormal findings (B + C + D type curves at least 2 times during the first 3 visits)	75%	87.5%	Mann–Whitney test 0.166
Audiometry: hearing loss at baseline	minimal = 50% moderate = 32% severe = 11%	minimal = 37% moderate = 43% severe = 0%	Chi-squared test 0.001
Parents’ initial report on nasal obstruction (abnormal = 2 and 3 on subjective scale 1–3)	96%	70%	Mann–Whitney test 0.006
Parents’ initial self-report on hearing (abnormal = 2 and 3 on subjective scales 1–3)	86%	77%	Mann–Whitney test 0.34
Prior number of acute otitis media/year/patient	1.6	1.5	Mann–Whitney test 0.46
Prior number of antibiotic treatments/year	1.6	1.7	Mann–Whitney test 0.71
Prior number of analgesic treatments/year	1.4	2.0	Mann–Whitney test 0.35
Indication for adenoidectomy present at baseline	7 out of 28 = 25%	27 out of 48 = 56.3%	Mann–Whitney test 0.01

**Table 4:** Results of primary outcome variables.

	2015	2020	*p*-Value
Frequency of antibiotic use, number of patients	5 out of 28 = 17.9%	10 out of 48 = 20.8%	Chi-squared test 0.753
Number of antibiotic treatments/year/patient	0.21	0.48	Mann–Whitney test 0.545
Use of analgesic or antipyretic medication, number of patients	6 out of 28 = 21.4%	16 out of 48 = 33.3%	Chi-squared test 0.27
Number of analgesic treatments/year/patient	0.36	0.63	Mann–Whitney test 0.257
Number of invasive surgical interventions	1 out of 28 = 3.6%	3 out of 48 = 6.25%	Mann–Whitney test 0.616
Improvement in tympanometric measurement with evaluation of the worse ear	A-type curve: normal pressure C- or D-type curve: under- or overpressure B-type curve: low admittance		See comparison in Figure 1a,b
Improvement in audiometric measurement with evaluation of the worse ear	Normal hearing: −10 to −20 dB Minimal hearing loss: −30 to −40 dB Middle hearing loss: −50 to −60 dB Severe hearing loss: >−60 dB		See the comparison in Figure 2

**Table 5:** Results of secondary outcome variables.

	2015	2020	*p*-Value
Number of acute otitis media during the observation period, count of patients	13 out of 28 = 46.4%	21 out of 48 = 43.8%	Chi-squared test 0.82
Number of acute otitis media/year/patient during the observation period	0.86	0.71	Mann–Whitney test 0.877
Adherence to prescribed therapies	1 (good) = 67.3 % 2 (intermediate) = 29.1% 3 (bad) = 3.6%	1 (good) = 56.2% 2 (intermediate) = 38.4% 3 (bad) = 5.4%	-
Average days between follow-up visits (visit 1–12), days, mean	49.4	48.7	Test for median differences and Mann–Whitney test 0.000
Length of observation period visit 1–12, days, mean	353	208	Mann–Whitney test 0.000
Adverse reactions	2%	2%	-

**Table 6:** Effect of different interventions in chronic otitis media with effusion (COME).

Reference	Mean Age (Years Range)	Diagnostic Method	Intervention	Time of Follow-Up (Months)	Effect
Rosenfeld 2016, 2003 [10,37]	NA (0–18)	NA	No intervention = Natural resolution	6 vs. 12	26% vs. 33%
Williamson 2015 [38]	5.4 (4–11)	Tympanometry, quality of life	Usual care alone ------------ Usual care plus autoinflation	1 vs. 3 ----------- 1 vs. 3	36% vs. 38% ----------- 47% vs. 50%
Bidarian-Moniri 2014 [39]	5 (2–8)	Tympanometry middle ear pressure (daPa) for both ears --------------- Audiometry mean, median hearing level (dB) for best ear	Balloon autoinflation	Inclusion 2 6 12 ------------ Inclusion 2 6 12	−389, −400 −189, −182 −196, −185 −151, −123 ------------ 22,22 16,13 16,13 14,12
Szőke [11]	4.7 (1–8)	Tympanometry, audiometry	P.E.A.N.U.T. method	12	70–80%
Chen 2020 [40]	NA	Audiometry (air-bone gap)	Surgery	6–18	80–90% (range 60–94)

## Data Availability

The data presented in this study are available on request from the corresponding author. The data are not publicly available due to privacy.

## Patents

The name “P.E.A.N.U.T. method^TM^” and the method itself have been trademarked. However, readers should feel free to use the method, either completely or any part of it. If cited or presented in literature, presentations, and media, it should be marked appropriately.

## References

[1] Zhang Y., Xu M., Zhang J., Zeng L., Wang Y., Zheng Q.Y. (2014). Risk Factors for Chronic and Recurrent Otitis Media—A Meta-Analysis. PLoS ONE.

[2] Rodney MDonlan J.W. (2002). Costerton Biofilms: Survival Mechanisms of Clinically Relevant Microorganisms. Clin. Microbiol. Rev..

[3] Chan C.L., Wabnitz D., Bardy J.J., Bassiouni A., Wormald P.-J., Vreugde S., Psaltis A.J. (2016). The microbiome of otitis media with effusion. Laryngoscope.

[4] Xu Q., Gill S., Xu L., Gonzalez E., Pichichero M. (2019). Comparative Analysis of Microbiome in Nasopharynx and Middle Ear in Young Children with Acute Otitis Media. Front. Genet..

[5] Barron C.L., Kamel-Abusalha L.B., Sethia R., Goodman S.D., Elmaraghy C.A., Bakaletz L.O. (2020). Identification of essential biofilm proteins in middle ear fluids of otitis media with effusion patients. Laryngoscope.

[6] Vanneste P., Page C. (2019). Otitis media with effusion in children: Pathophysiology, diagnosis, and treatment. A review. J. Otol..

[7] Wallace I.F., Berkman N.D., Lohr K.N., Harrison M.F., Kimple A.J., Steiner M.J. (2014). Surgical treatments for otitis media with effusion: A systematic review. Pediatrics.

[8] Hao J., Chen M., Liu B., Yang Y., Liu W., Ma N., Han Y., Liu Q., Ni X., Zhang J. (2019). Compare two surgical interventions for otitis media with effusion in young children. Eur. Arch. Otorhinolaryngol..

[9] Yin G., Tan J., Li P. (2019). Balloon dilation of Eustachian tube combined with tympanostomy tube insertion and middle ear pressure equalization therapy for recurrent secretory otitis media. J. Otol..

[10] Rosenfeld R.M., Shin J.J., Schwartz S.R., Coggins R., Gagnon L., Hackell J.M., Hoelting D., Hunter L.L., Kummer A.W., Payne S.C. (2016). Clinical Practice Guideline: Otitis Media with Effusion Executive Summary (Update). Otolaryngol. Head Neck Surg.

[11] Szőke H., Maródi M., Sallay Z., Székely B., Sterner M.G., Hegyi G. (2016). Integrative versus Conventional Therapy of Chronic Otitis Media with Effusion and Adenoid Hypertrophy in Children: A Prospective Observational Study. Forsch. Komplementmed..

[12] Tavernier L., Chobaut J.-C. (2006). Eustachian tube rehabilitation therapy: Indications, techniques, and results. Fr ORL.

[13] Leunig A., Mees K. (1995). Middle ear ventilation with the Otovent latex membrane system. Laryngo Rhino Otol..

[14] Blanshard J., Maw A., Bawden R. (1993). Conservative treatment of otitis media with effusion by autoinflation of the middle ear. Clin. Otolaryngol. Allied Sci..

[15] Stangerup S., Sederberg-Olsen J., Balle V. (1991). Treatment with the Otovent device in tubal dysfunction and secretory otitis media in children. Ugeskr. Laeger.

[16] Lee H.J., Park S.K., Choi K.Y., Park S.E., Chun Y.M., Kim K.S., Park S.N., Cho Y.S., Kim Y.J., Kim H.J. (2012). Korean clinical practice guidelines: Otitis media in children. J. Korean Med. Sci..

[17] Juntti H., Tikkanen S., Kokkonen J., Alho O.P., Niinimäki A. (1999). Cow’s milk allergy is associated with recurrent otitis media during childhood. Acta Otolaryngol..

[18] Baars E.W. (2012). A mixed-method approach in reviewing the effects, safety and working principles of Citrus/Cydonia on hay fever. Eur. J. Integr. Med..

[19] Gründemann C., Papagiannopoulos M., Lamy E., Mersch-Sundermann V., Huber R. (2011). Immunomodulatory properties of a lemon-quince preparation (Gencydo^®^) as an indicator of anti-allergic potency. Phytomedicine.

[20] Baars E.W., Savelkoul H.F.J. (2008). Savelkoul Citrus/Cydonia Comp. Can Restore the Immunological Balance in Seasonal Allergic Rhinitis-Related Immunological Parameters In Vitro. Mediat. Inflamm..

[21] Baars E.W., Jong M.C., Boers I., Nierop A.F.M., Savelkoul H.F.J. (2012). A Comparative In Vitro Study of the Effects of Separate and Combined Products of Citrus e fructibus and Cydonia e fructibus on Immunological Parameters of Seasonal Allergic Rhinitis. Mediat. Inflamm..

[22] Fattouch S., Caboni P., Coroneo V., Tuberoso C.I., Angioni A., Dessi S., Marzouki N., Cabras P. (2007). Antimicrobial Activity of Tunisian Quince (Cydonia oblonga Miller) Pulp and Peel Polyphenolic Extracts. J. Agric. Food Chem..

[23] Baars E.W., Jong M.C., Nierop A.F.M., Boers I., Savelkoul H. (2011). Subcutaneous Injections Versus Nasal Spray for Seasonal Allergic Rhinitis: A Randomized Controlled Trial on Efficacy and Safety. ISRN Allergy.

[24] Fattouch S., Caboni P., Coroneo V., Tuberoso C.I., Angioni A., Dessi S., Marzouki N., Cabras P. (2016). Huber Efficacy of a Nasal Spray from Citrus limon and Cydonia oblonga for the Treatment of Hay Fever Symptoms—A Randomized, Placebo Controlled Cross-Over Study. Phytother. Res..

[25] Huber R., Stintzing F.C., Briemle D., Beckmann C., Meyer U., Gründemann C. (2011). In Vitro Antiallergic Effects of Aqueous Fermented Preparations from Citrus and Cydonia fruits. Planta Med..

[26] Imenshahidi M., Hosseinzadeh H. (2016). Berberis Vulgaris and Berberine: An Update Review. Phytother. Res..

[27] Vagedes J., Helmert E., Kuderer S., Müller V., Voege P., Szőke H., Valentini J., Joos S., Kohl M., Andrasik F. (2018). Effects of Footbaths with Mustard, Ginger, or Warm Water Only on Objective and Subjective Warmth Distribution in Healthy Subjects: A Randomized Controlled Trial. Complementary Ther. Med..

[28] Venekamp R., Burton M.J., Van Dongen T.M.A., Van Der Heijden G.J., Van Zon A., Schilder A.G.M. (2016). Antibiotics for otitis media with effusion in children. Cochrane Database Syst. Rev..

[29] Grijalva C.G., Nuorti J.P., Griffin M.R. (2009). Antibiotic prescription rates for acute respiratory tract infections in US ambulatory settings. JAMA.

[30] Hamre H.J., Fischer M., Heger M., Riley D., Haidvogl M., Baars E.W., Bristol E., Evans M., Schwarz R., Kiene H. (2005). Anthroposophic vs. conventional therapy of acute respiratory and ear infections. Wien. Klin. Wochenschr..

[31] Hamre H.J., Glockmann A., Schwarz R., Riley D.S., Baars E.W., Kiene H., Kienle G.S. (2014). Antibiotic Use in Children with Acute Respiratory or Ear Infections: Prospective Observational Comparison of Anthroposophic and Conventional Treatment under Routine Primary Care Conditions. Evid. Based Complement. Altern. Med..

[32] Baars E.W., Zoen E.B.-V., Breitkreuz T., Martin D., Matthes H., Von Schoen-Angerer T., Soldner G., Vagedes J., Van Wietmarschen H., Patijn O. (2019). The Contribution of Complementary and Alternative Medicine to Reduce Antibiotic Use: A Narrative Review of Health Concepts, Prevention, and Treatment Strategies. Evid. Based Complement. Altern. Med..

[33] Jeschke E., Lüke C., Ostermann T., Tabali M., Hübner J., Matthes H. (2007). Prescribing practices in the treatment of upper respiratory tract infections in anthroposophic medicine. Forsch. Komplementärmedizin.

[34] MacKay D. (2003). Can CAM therapies help reduce antibiotic resistance? (Antibiotic Resistance). Altern. Med. Rev..

[35] Sarrell E.M., Cohen H.A., Kahan E. (2003). Naturopathic treatment for ear pain in children. Pediatrics.

[36] Levi J.R., Brody R.M., McKee-Cole K., Pribitkin E., O’Reilly R.C. (2013). Complementary and alternative medicine for pediatric otitis media. Int. J. Pediatric Otorhinolaryngol..

[37] Rosenfeld R.M., Kay D. (2003). Natural history of untreated otitis media. Laryngoscope.

[38] Williamson I.G., Vennik J., Harnden A., Voysey M., Perera R., Kelly S., Yao G., Raftery J., Mant D., Little P. (2015). Effect of nasal balloon autoinflation in children with otitis media with effusion in primary care: An open randomized controlled trial. CMAJ.

[39] Bidarian-Moniri A., Ramos M.-J., Ejnell H. (2014). Autoinflation for treatment of persistent otitis media with effusion in children: A cross-over study with a 12-month follow-up. Int. J. Pediatric Otorhinolaryngol..

[40] Chen S., Zhao M., Zheng W., Wei R., Zhang B., Tong B., Qiu J. (2020). Myringotomy and tube insertion combined with balloon eustachian tuboplasty for the treatment of otitis media with effusion in children. Eur. Arch Otorhinolaryngol..

